# Cell-Based Sensor System Using L6 Cells for Broad Band Continuous Pollutant Monitoring in Aquatic Environments

**DOI:** 10.3390/s120303370

**Published:** 2012-03-08

**Authors:** Rebekka Kubisch, Ulrich Bohrn, Maximilian Fleischer, Evamaria Stütz

**Affiliations:** 1 Pharmaceutical Biology-Biotechnology, Department of Pharmacy, Center for Drug Research, Ludwig-Maximilian-University Munich, Butenandstraße 5-13, Building D, 81377 Munich, Germany; E-Mail: rebekka.kubisch@cup.uni-muenchen.de; 2 Corporate Research & Technologies, Siemens AG, Otto-Hahn-Ring 6, 81739 Munich, Germany; E-Mails: ulrich.bohrn.ext@siemens.com (U.B.); maximilian.fleischer@siemens.com (M.F.)

**Keywords:** cell-based sensor, whole-cell sensor, cytotoxicity sensor, online water monitoring, impedance, acidification, respiration, heavy metals, nicotine, acetaminophen

## Abstract

Pollution of drinking water sources represents a continuously emerging problem in global environmental protection. Novel techniques for real-time monitoring of water quality, capable of the detection of unanticipated toxic and bioactive substances, are urgently needed. In this study, the applicability of a cell-based sensor system using selected eukaryotic cell lines for the detection of aquatic pollutants is shown. Readout parameters of the cells were the acidification (metabolism), oxygen consumption (respiration) and impedance (morphology) of the cells. A variety of potential cytotoxic classes of substances (heavy metals, pharmaceuticals, neurotoxins, waste water) was tested with monolayers of L6 cells (rat myoblasts). The cytotoxicity or cellular effects induced by inorganic ions (Ni^2+^ and Cu^2+^) can be detected with the metabolic parameters acidification and respiration down to 0.5 mg/L, whereas the detection limit for other substances like nicotine and acetaminophen are rather high, in the range of 0.1 mg/L and 100 mg/L. In a close to application model a real waste water sample shows detectable signals, indicating the existence of cytotoxic substances. The results support the paradigm change from single substance detection to the monitoring of overall toxicity.

## 1. Introduction

The World’s fresh water resources are facing drastically increasing pollution with numerous toxic substances harmful to organic life [1]. These aquatic system pollutants are mainly man-made, due to industrialization and intensive farming. In Europe, huge efforts have been undertaken from the governmental side in form of regulations (e.g., Water Framework Directive (WFD) [2]) to control and inhibit any further increases in water pollution. The WFD defines a number of biological and chemical parameters that should be part of broad range monitoring programs in the near future with a focus on 33 priority chemical substances (including heavy metals like nickel, lead and mercury) (see Table 1).

Qualitative and quantitative detection of inorganic and organic substances is classically done by expensive chromatographic [3] and spectroscopic [4], as well as standard wet chemistry methods (e.g., pH, hardness, salinity, biological oxygen demand, *etc.*). Unfortunately, these standard analytical chemical methods do not supply direct information about the overall toxicity or bioactivity of water sources. The evaluation of the toxicity is highly favored as there is an uncountable number of sometimes unexpected potential pollutants which can not be qualitatively detected with standard methods. Especially organic pollutants can have a high adverse bioactivity, even in very low concentrations. Another disadvantage is the time dependent nature of these methods, normally consisting of an intermittent sampling, separation, device calibration, measurement and data evaluation, thus being unsuitable for continuous monitoring of water. To fulfill the proposed criteria of regulations like the WFD, water in batch and flow systems has to be intensively controlled, especially in waste water treatment plants and in water distribution networks. A significant amount of conventional water pollution tests would result in enormous financial costs which are hard to handle, especially in developing countries which face even severer problems concerning polluted water [5].

The use of chemical sensors and biosensors for the environmental monitoring of water is very common [6,7]. Most of these techniques are based on optical, electrochemical and on immunological detection methods. Seidel *et al.* [8] recently reviewed the use of microarray techniques using nucleic acid biosensors for the parallel detection of multiple analytes (toxins, endocrine-disrupting compounds, pesticides) regarding applications in the field of water monitoring. Inhibition of enzymes (e.g., amidases, esterases, dehydrogenases or kinases) is used for the application of a conductometric measurement method presented by Jaffrezic-Renault *et al.* [9] to detect different heavy metal ions, pesticides and herbicides. In another review by Namour *et al.* [10], water monitoring regarding the inorganic priority substances (Cd, Hg, Ni, Pb) of the WFD using microsensors was investigated in detail. Palchetti *et al.* [11] gave an overview of advances in the development and applications of nucleic acid-based biosensors with focus on functional nucleic acid elements and the detection of DNA damage induced by genotoxic pollutants, solvents, polycyclic aromatic hydrocarbons and pesticides. A severe problem in environmental water monitoring is the diverse organic carbon level. Tschmelak *et al.* [12] confronted this problem with an ultrasensitive immunoassay for estrone quantification using the optical immunosensor RIANA.

Bioassays for toxicity detection based on fish [13], water fleas [14] or algae [15] are routinely used for the monitoring of water. Though the use of toxicity bioassays are often still time-intensive and this makes them also not always the first choice for an online monitoring technique.

The use of *in vitro* methods in water toxicology research has a long tradition [16]. One major drawback is the lack of the possibility of online monitoring because most *in vitro* assays are time consuming and laborious. These endpoint assays provide a lot of high specific information. Concerning water quality monitoring, one might be more interested in continuous information about the overall toxicity and the adverse effect on humans, rather than qualitative and quantitative data of the contaminant itself. In the last decades, several whole-cell based sensor systems have been developed for the monitoring of water appearing as complementary and perhaps advantageous techniques to standard biological and chemical methods [17–20].

The use of bacteria-based biosensors for ecotoxicology testing is quite common as they represent the majority of cell-based sensors [21]. For example, genetically modified bacteria express luminescent products when pollutants are present. These types of sensors are used in activated sludge treatment facilities for the monitoring of the respiration activity and the organic pollution in the effluent of a wastewater treatment plant [22]. One great merit of many microbial based biosensors up to now is the ability to classify different kinds of toxicity with multi-channel systems [23,24]. The systems and reporter constructs developed in the field so far have the capability to distinguish between DNA damage, oxidative damage, heavy metals, endocrine disrupting compounds, aromatic organic solvents, genotoxicants *etc.* which allows for a sort of fingerprinting of the water pollutants [25–27]. Bacteria-based biosensors for water quality monitoring [28,29] have to deal thoroughly with the immobilization or encapsulation of the microorganisms, otherwise there is the possibility of a washing-out of the bacteria which could lead to contaminations itself, if the biosensor is implemented online in the water delivery network.

In contrast to bacteria, mammalian cell lines are supposed to mimic the physiology of the human body better than bacteria or yeast. These cells are easy to cultivate, provide information about the bioavailability and the toxicity of the pollutants towards eukaryotic cells. Eukaryotic cell-based sensors are mainly developed for a usage in the medical field, rather than for environmental purposes. For the application as a sensor for water monitoring, the use of eukaryotic cells might have some advantages. Eukaryotic cells have no cell wall and are therefore supposed to take up especially larger toxic substances easier and faster through their cell membrane. With the ability to adhere on substrates without further immobilization, eukaryotic cells enable the use of impedance electrodes for cell characterization.

One initial technology utilizing living adherent eukaryotic cells for the detection of toxic substances is the electrical-cell-impedance-system (ECIS) [30,31] Over the last decades, many groups have investigated the ability of impedance based methods [32] for the detection of pollutions in aqueous environments. Recent studies figured out that nine out of 12 waterborne industrial chemicals (e.g., copper, nicotine, ammonia) can be detected by two cell lines (BLMVEC, IgH-2) using cell impedance technology [33,34]. The value of impedance only as the best readout parameter, however, appears to be subject to several discussions and questions about the benefit of more powerful sensor parameters arise.

One of the most versatile cell-based sensor systems on the market is the Bionas Analyzing System (Bionas, Rostock, Germany). Its main benefit is the supply of three cellular readout-parameters (cell impedance [35,36], acidification, respiration [37]). These can be detected in parallel and continuously over a long time. The device was originally used in pharmaceutical research for drug testing [38], for the optimization of culture conditions [39] but also in environmental areas, not only for the monitoring of water [40], but also for the monitoring of air and the detection of toxic gases [41–43].

Eukaryotic cells have to fulfill certain criteria for their use as chemical sensors in the water monitoring:

- High sensitivity to various compounds
- No or low proliferation
- Long lifespan (weeks to month)
- Unchanged sensitivity over a long period of time
- Rapid response
- Genetic stability
- Easy to handle
- Stable and low-noise signals during measurement
- Moderate costs

High selectivity is (although valuable) not primarily intended as the system should give information about the presence of any toxic substance. A low selectivity compared with high sensitivity makes such a system appropriate for the detection of a wide range of unknown toxic substances. One benefit of cell sensors is their ability to react only to the bioavailable fraction of metal ions. In contrast, standard analytical methods are not able to distinguish between bioavailable and non-bioavailable fractions of metals [44].

Cell-free sensor methods as described above are in many cases more robust and easier to handle. However, the big advantage of the cell-based methods is the fact, that the cell as a detection entity reacts towards a lot more substances and environmental changes than cell-free sensors which are in general focused on the detection of single substances with high accuracy. The use of both microbial and eukaryotic cells is relatively cost effective, compared to the use of highly purified antibodies for immunoassay.

The aim of this study was to investigate the applicability of a multiparametric cell-based sensor technology for the real-time monitoring of water. The growth characteristics and sensitivity of different commercially available cell lines towards the exposure of waterborne industrial chemicals was investigated. The chosen cell lines were evaluated regarding their performance in terms of signal stability (low background noise), growth speed, sensitivity (but not selectivity), availability, manageability and long term stability. The most promising cell line was used for the toxicity testing of a variety of different substance classes (heavy metals, pharmaceuticals, pesticides).

## 2. Experimental Section

### 2.1. Cell Culture

V79 (Chinese hamster lung fibroblasts) and HT-29 (human colon ardenocarcinoma) cells were purchased from DSMZ (German Collection of Microorganisms and Cell Lines, Braunschweig, Germany), L6 (rat skeletal muscle) and HepG2 (liver hepatocellular carcinoma) cells were purchased from ATCC (American Type Culture Collection, Manassas, VA, USA), NHDF (normal human dermal fibroblasts) cells were purchased from Promocell (Heidelberg, Germany) and primary canine hepatocyte cells were purchased from Primacyt (Schwerin, Germany). All cell lines were grown at 37 °C in 5% CO_2_ in a humidified atmosphere. V79, L6, NHDF, and HepG2 cells were cultivated in Dulbecco’s modified Eagle medium (DMEM; Gibco, Darmstadt, Germany), supplemented with 10% heat inactivated fetal calf serum (FCS; Biochrom, Berlin, Germany), 100 units/mL penicillin (BioWhittaker, Heidelberg, Germany) and 100 μg/mL streptomycin (BioWhittaker). HT-29 cells were cultivated in McCoy’s medium (Invitrogen, Darmstadt, Germany), supplemented with 10% heat inactivated FCS, 100 Units/mL penicillin and 100 μg/mL streptomycin. Primary canine hepatocyte cells were cultivated in modified DMEM [45] supplied with 10% FCS, Hepes (1 mM), sodium bicarbonate (0.05 mM), nicotinamide (305 mg/L), acetylcysteine (5 mM) and ascorbic acid (0.2 mM).

### 2.2. Chemicals

Nickel chloride (NiCl_2_), copper sulfate (CuSO_4_), acetaminophen were purchased from Sigma Aldrich (Steinheim, Germany). Nicotine was purchased from Merck (Darmstadt, Germany). A fresh waste water sample was kindly provided by the Bavarian Environment Agency.

### 2.3. Bionas Analyzing System

**Figure 1.**
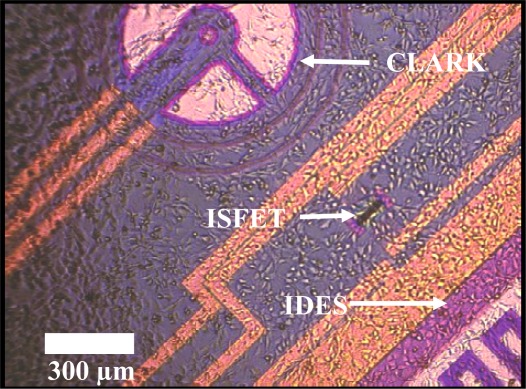
Adherent monolayer of L6 (rat skeletal muscle cells) cells on a Bionas SC 1000 Metabolic Chip.

The system has been described in detail elsewhere [46]. In brief, the experimental setup was as follows: depending on the size of the cells, (2–10) × 10^5^ cells per chip (SC 1000 Metabolic chip, Bionas, see Figure 1) were seeded 1 day before the start of the experiment and cultured in growth media in a 5% CO_2_ atmosphere at 37 °C under saturated humidity to form a confluent cell monolayer within 24 h. The growth area on the chip which is defined by the chip housing is 70.88 mm^2^. One hour prior to inserting the chips into the Bionas System device, growth medium on the chips was replaced by carbonate buffer-free running medium containing 1 mM Hepes (BioWhittaker) instead and reduced (1%) serum concentration.

**Figure 2.**
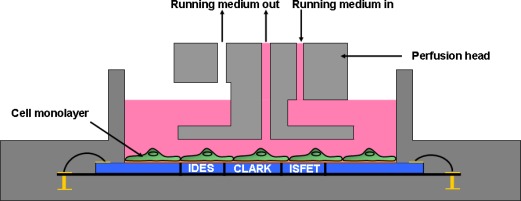
Schematic cross section of a SC 1000 Metabolic Chip (Bionas) with the perfusion head of the Bionas analyzing system device. The silicone chip (blue) incorporates three types of electrodes: impedance electrode (IDES), oxygen electrode (CLARK) and pH electrode (ISFET).

The metabolic chips were placed into the six parallel biomodules of the Bionas Analyzing System device. In a stop/go-modus (3–4 min interval), fresh medium (without contaminants) was pumped over the cells through a perfusion head (see Figure 2) via the fluidic system with a gentle flow rate of 56 μL per minute during the go-phase.

With this pumping speed, the cell monolayer is not affected and the cells are still able to adhere on the sensor surface. An interval of 3–4 min was chosen to generate as many data points as possible and to ensure that the whole medium covering the cells during the stop phase (120 μL), is exchanged completely. The data of the three different parameters (pH, O_2_, impedance) of each sensor chip were acquired. During the 3–4 min stop-phase, the slope of the acidification signals as well as the slope of the respiration signals were recorded and displayed in a standardized form. Every 6 min (3 min stop–3 min go) or 8 min (4 min stop–4 min go), respectively, one data point is plotted in the standardized diagram. The buffer capacity of the running medium is rather low (1 mM Hepes) which enables to measure subtle changes in the total acidification during 3–4 min. The impedance signal is measured every 10 s at 10 kHz with two palladium interdigitated electrode structures (IDES). Oxygen consumption measurements are performed with two Clark-type electrodes while the acidification is measured with five ion selective field effect transistors (ISFETs, Al_2_O_3_). Both electrode types have an Ag/AgCl reference electrode which is located in the medium outlet of the perfusion head. Test substances like NiCl_2_, CuSO_4_, nicotine and acetaminophen were diluted in running medium to obtain the test substance solutions. After an adaption phase of at least three hours without contamination, the test substance solutions were applied to the cells for 14 h. One channel was run without contaminations to serve as a reference; the five other were subjected to different levels of contamination. To standardize the recorded raw data one specific data point obtained during the adapting period (after three hours of adaption) was chosen and set as 100%. All three parameters were standardized to this value. After each exposure period, a recovery phase of at least two hours was added to figure out, whether the cells are able to recover from the substance exposure. At the end of each experiment, a solution containing 0.2% Triton X-100 (TX) was added to terminate the cells and generate a baseline of the sensor signals without cells. In order to figure out the characteristics of the cell growth behavior, the cells were constantly supplied with untreated running medium for 18 h.

For the testing of the water sample, the undiluted water sample was first sterile filtered. Afterwards, dry powdered DMEM (Sigma) containing all nutrients and constituents that are part of the standard liquid medium, were dissolved with the sterile filtered water samples. Further dilutions (1:10, 1:100, 1:1,000, and 1:10,000) were generated by adding fresh running medium (solution form). To avoid any unspecific cellular reaction, the osmolarity and the pH of the solution were adjusted appropriately.

### 2.4. BrdU Proliferation Assay

A cell proliferation kit (Roche Diagnostics GmbH, Mannheim, Germany) was used to determine the amount of 5-bromo-20-deoxyuridine (BrdU) incorporated in the DNA. The assay was performed according to the manufacturer’s instructions. Briefly, 2 × 10^4^ L6 cells per well were seeded in black, clear bottom 96 well plates (Corning, New York, NY, USA). 24 h after seeding media was changed to 1% FCS and cells were treated with either vehicle control or different concentrations of test substances nicotine or nickel for 24 h. Afterwards the cells were incubated for 2 h at 37 °C with 100 mM BrdU. The cells were fixed and anti-BrdU-POD (monoclonal antibody from mouse conjugated with peroxidase) was added, which binds to the incorporated BrdU in the newly synthesised DNA. The immune complex was detected by the subsequent substrate reaction (tetramethylbenzidine) and the absorbance of the blue-green reaction product was measured at 370 nm with a microtiter plate reader (FLUOstar OPTIMA, BMG Labtech, Ortenberg, Germany).

## 3. Results and Discussion

### 3.1. Experimental Evaluation of Cell Cultures

Cells, acting as sensitive layers in cell-based sensor technology, have to fulfill a number of characteristics. One key characteristic is the generation of stable signals without a high noise background level. The shape of the signal curve should be constant and reproducible. At the time when a confluent monolayer is established, the cells must not overgrow each other as this would lead to an increase in the total acid production and oxygen consumption. Therefore, the cell growth should be relatively slow. From a more practical point of few, the cells should be robust and easy to handle and maintain, even by minor trained workers. A stable genetic background (which can not be assured with a cancer cell line) is as well favored as an easy availability.

Cell lines are characterized in many ways in the scientific literature, e.g. growth speed, genetic stability, expression profiles, doubling time *etc.* But only few data exist that explain the behavior of cell lines in fluidic systems on a silicone chip. The intention of this evaluation was to generate experience of the robustness of different types of cells towards the mechanical stress in the fluidic systems. The commercially available cell lines were chosen to cover huge variety of cell lines from different origins ranging from primary cells, liver cells, cancer cells and human cells.

Evaluation of the six chosen cell types was done by seeding cells on the metabolic chips. Their metabolic characteristics were assessed using the Bionas Analyzing System. All cell types settled down and attached easily to the silicon surface of the sensor chip. The ability to adhere at the sensor surface is crucial as the signal generation is highly influenced by the spatial vicinity of the cells. Fresh seeded cells sink to the ground until they get in contact with the sensor surface. The eukaryotic cells express multiple adhesion molecules which mediate the anchoring process to the substrate. No additional immobilization or fixation is needed as the cells form a stable cell monolayer on the chip surface.

**Figure 3.**
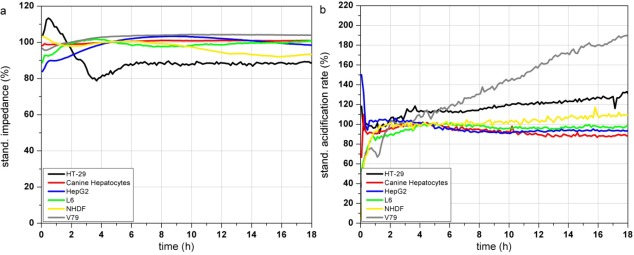

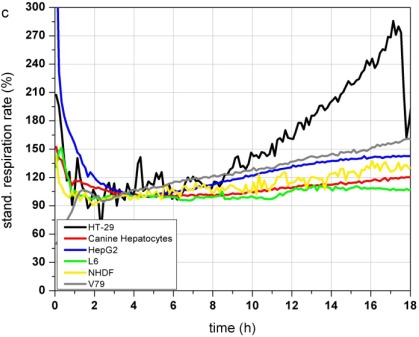
Time course of impedance (**a**), acidification (**b**) and respiration (**c**) of different eukaryotic cell types during 18 h incubation in the Bionas 2500 Analyzing System: human colon carcinoma cell line HT-29 (black), Canine hepatocytes (red), hepatocellular carcinoma cell line HepG2 (blue), rat skeletal muscle cell line L6 (green), normal human dermal fibroblasts NHDF (yellow) and Chinese hamster lung fibroblasts V79 cells (grey).

Figure 3(a–c) show the response profiles of the tested cell types summarized in Table 2. Changes in the adhesion properties or in the cellular morphology assessed by the impedance measurement do not occur over at least 18 h of cultivation in any of the cells (Figure 3(a)).

The metabolic parameters (acidification and respiration) were stable in five of the tested cell types (HT-29, canine hepatocytes, HepG2, L6 and NHDF) measured by the acidification rate. V79 cells showed an increasing acidification rate (Figure 3(b)) starting right at the beginning of the test. The respiration rate of canine hepatocytes, HepG2, L6 and NHDF cells kept stable over the whole 18 h of culturing. In contrast HT-29 cells showed increasing respiration activity even after 9 h (Figure 3(c)). The respiration rate of V79 cells increased constantly from the start of the monitoring-process. The slope of the increasing respiration curve is comparable to their acidification rate. HepG2 showed a moderately increasing respiration rate up to 145% after 18 h.

The comparison of the six cells types showed that the fast growing colon carcinoma cell line HT-29 and Chinese hamster fibroblast cell line V79 do not show suitable characteristics for the use as a sensitive layer in the Bionas technology for long time monitoring of water quality (see Table 2). Due to their fast growth acidification and respiration rates strongly increase even without treatment. Also, primary cell isolates (like the canine hepatocytes) can be grown on the metabolic chips. But even if their metabolic characteristics would meet the criteria of stable signals and slow cell growth, their properties may differ from donor to donor. Another disadvantage of primary isolates is their limited lifespan. Hepatocellular carcinoma HepG2 cells showed stable signals during the on-chip culturing process but proved a low sensitivity to different model substances in former experiments as well as a low reproducibility between several passages and a genetic instability (data not shown). The rat myoblast cell line L6 has proven to be suitable for this approach because they showed stable signals during the on chip cultivation. Nevertheless the cells are easy to handle as they are a commercially available cell line with low media requirements. Stable signals occur since L6 cells are muscle precursor cells and stop proliferation when reaching confluence, then they start to differentiate into myotubes (muscle cells). Regarding their stable signals and their easy maintenance, L6 cells were chosen as a model cell line for further experiments with test substances. Unfortunately, changes between single control measurements, without the treatment with toxic substances, can not be avoided at all, as the time course depends on many parameters (e.g., pH, osmolarity, temperature, medium composition, number of passage, *etc.*). A major factor for alterations between individual control measurements is the seeding density. If the number of cells, which are seeded the day before the experiment, is too low, there is still free area on the sensor surface. Then, the cells have enough space to grow and divide which would result in a constant increase of the cellular parameters, until a plateau is reached and further cell growth is inhibited. Therefore, the time course of the control measurements itself provides information of the quality of the single measurement.

### 3.2. Real-Time Monitoring of Cellular Response towards Pollutants

Four cytotoxic substances (artificial pollutants) and one real waste water sample were tested to show the response behavior of the chosen cell Line (L6) towards these compounds. The compounds represent different types of potential contaminants including inorganic compounds (metals), pharmaceuticals and neurotoxins.

#### 3.2.1. Nickel Chloride (NiCl_2_)

Nickel is one of the 33 priority chemical substances of the WFD list (see Table 1). It induces oxidative stress but is only mildly active in reactive oxygen species (ROS) induction compared to other metal species like chrome and cadmium [47]. Nickel is known to decrease rapidly mitochondrial activity and cell viability in primary and passaged cells at concentrations above 100 μM [48]. Beside acute toxic effects of nickel compounds, it is also known for its genotoxic, immunotoxic, neurotoxic, reproductive toxic and carcinogenic potential [49,50]. Oxidative damage induced by nickel is caused due to generation of reactive oxygen species (ROS) by a Fenton-type reaction, or by inactivated enzymes involved in the cellular defenses against reactive oxygen systems [51]. Further on, *in vitro* studies revealed that exposure to soluble nickel species results in a disruption of the epithelial barrier function observed by alterations in transepithelial electrical resistance [52].

**Figure 4.**
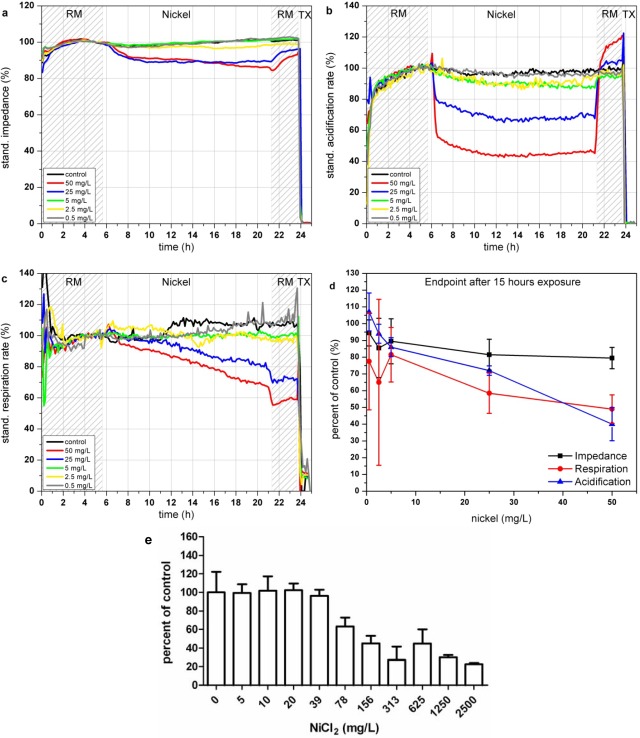
Time course of the impedance (**a**), acidification (**b**) and respiration (**c**) of L6 rat skeletal muscle cells during 24 h incubation with NiCl_2_ in the Bionas 2500 Analyzing System. Vehicle control (black), 50 mg/L (red), 25 mg/L (blue), 5 mg/L (green), 2.5 mg/L (yellow) and 0.5 mg/L (grey). Phases with running medium (RM) represent culture medium treatment without added substances. (**d**) Percent of control of impedance, respiration and acidification endpoint values measured after 15 h of nickel chloride exposure (n = 3). (**e**) Results of BrdU assay of L6 cells incubated with nickel chloride for 24 h.

In the Bionas analyzing system, the presence of nickel ions results in a decrease of impedance signals of L6-cells down to almost 80% at concentrations of 25 and 50 mg/L (see Figure 4(a)) which might arise from the membrane disruptive effects of nickel ions. The acidification is immediately impaired at the beginning of the incubation with Ni^2+^-containing media solution (see Figure 4(b)). If Ni^2+^ ions are removed cells are able to recover from this impact within three hours also after exposure to 50 mg/L Ni^2+^. A low nickel concentration of 0.5 mg/L seems to slightly activate the cellular metabolism, indicated in an increase of extracellular acidification (see Figure 4(d)). Similar to the acidification, the cellular respiration is inhibited only at high Ni^2+^ concentrations (25 and 50 mg/L; see Figure 4(c)).

Compared to the acidification rates cells are not able to recover a normal respiration rate after Ni^2+^ removal, as the respiration rate remained low in the recovery phase at the end of the experiments. The highest tested nickel concentrations of 50 mg/L reduce the respiration during 15 h of exposure down to 60%. The reported impairment of mitochondrial activity as well as the generation of ROS caused by nickel ions seems to be possible explanations for the lasting reduction of cellular respiration. The endpoint values of the changes relative to the control after 15 h of incubation with the various nickel concentrations are shown in Figure 4(d). The results achieved by the BrdU assay show that proliferation inhibition starts in the range between 20 and 78 mg/L Ni^2+^ which is in good agreement with the data achieved with the Bionas analyzing system (see Figure 4(e) and Table 3). Results comparable to those of the BrdU assay within 5–7 h could be achieved only by the observation of the acidification.

#### 3.2.2. Copper Sulfate (CuSO_4_)

Copper is well known for its cytotoxic effects as it is a redox active metal and possesses the ability to produce reactive radicals such as superoxide anion radicals (via Fenton reaction) and nitric oxide in biological systems [54]. As a consequence, this oxidative stress leads to lipid peroxidation, DNA damage and protein modifications. Copper entering the human body via diet (∼2 mg/day) and also the consumption of drinking water out of copper tubing is known to have adverse health effects for humans, as there is always a constant amount of copper ions eluted into the drinking water. Copper sulfate solutions were heavily used as a fungicide in vineyards (so called Bordeaux mixture) to control downy mildew [55].

**Figure 5.**
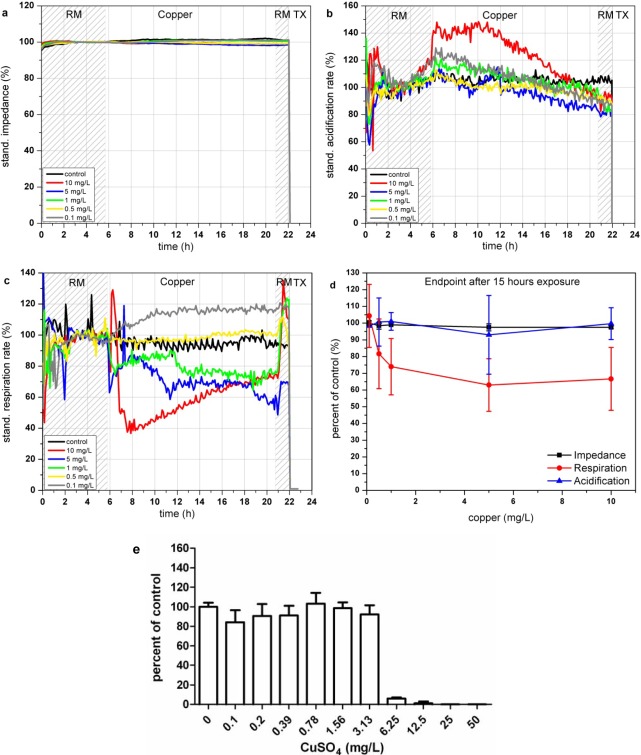
Time course of the impedance (**a**), acidification (**b**) and respiration (**c**) of L6 rat skeletal muscle cells during 24 h incubation with CuSO_4_ in the Bionas 2500 Analyzing System. Vehicle control (black), 10 mg/L (red), 5 mg/L (blue), 1 mg/L (green), 0.5 mg/L (yellow) and 0.1 mg/L (grey). Phases with running medium (RM) represent culture medium treatment without added substances. (**d**) Percent of control of impedance, respiration and acidification endpoint values measured after 15 h of copper sulfate exposure (n = 3). (**e**) Results of BrdU assay of L6 cells incubated with copper sulfate for 24 h.

In the Bionas analyzing system, copper sulfate shows nearly no effect on the impedance signals of the L6 cells (see Figure 5(a)). In contrast the cellular metabolism is heavily altered by the copper ions which results in elevated acidification rates only at the beginning of the incubation at a concentration of 10 mg/L. Also other concentrations lower than 10 mg/L show a small increase in the acidification, compared to the untreated control measurements. Interestingly, the acidification rates decrease after this first increase (3 h after copper addition). The exposure to copper might lead to a first activation of the metabolism which is followed by another cellular response, tuning down the glycolysis (see Figure 5(b)). The cellular respiration rate is decreased at concentrations larger than 0.5 mg/L (see Figure 5(c)) which is supposedly caused by the oxidative stress due to reactive radicals. The respiration rates are able to recover at least partially after removal of copper sulfate containing media at the end of the experiment. Low concentrations of about 0.1 mg/L lead to a slight increase (Figure 5(d)).

The relative changes compared to the control measurement demonstrate, that only the observation of the respiration can indicate the presence of copper ions in concentrations higher than 1 mg/L (see Figure 5(d) and Table 3). The endpoint values of the changes relative to the control after 15 h of incubation with the various copper concentrations are shown in Figure 5(d). BrdU assay results indicate a cytotoxic effect starting at concentrations between 0.78 and 6.25 mg/L copper ions (see Figure 5(e)). A similar concentration range for the start of cytotoxic copper concentrations as well as a fast reduction of viability can be achieved by the observation of the oxygen consumption parameter of the sensor system.

#### 3.2.3. Acetaminophen (AAP)

Acetaminophen (AAP, paracetamol, C_8_H_9_NO_2_) is one of the most widely used pain reliever drugs and one of the first described examples of bioactivation-dependent toxicity [56]. Due to its high popularity as an antipyretic drug it is one of the highest volume production pharmaceuticals and therefore represents the main pollutant of a pharmaceutical waste water and is reported as one of the most frequently detected pharmaceuticals in sewage treatment plant effluents, drinking water, or surface water [57–59]. The measured levels of acetaminophen were much lower than the predicted values [60] which may reflect rapid degradation characteristics [61] of acetaminophen.

Cytochrome P450 isoenzymes convert a minor part of paracetamol to a highly-reactive intermediate metabolite, *N*-acetyl-*p*-benzoquinoneimine (NAPQI). NAPQI is then irreversibly conjugated with the sulfhydryl groups of glutathione. Generally, NAPQI is detoxified by conjugation with glutathione to form cysteine and mercapturic acid conjugates. In cases of a paracetamol overdose, more paracetamol is shunted to the cytochrome P450 system to produce NAPQI. As a consequence, glutathione becomes exhausted because the demand for glutathione is higher than its regeneration. NAPQI therefore remains in its toxic form in the cell and reacts with cellular membrane molecules, resulting in severe cell damage and leading to cell death [62].

**Figure 6.**
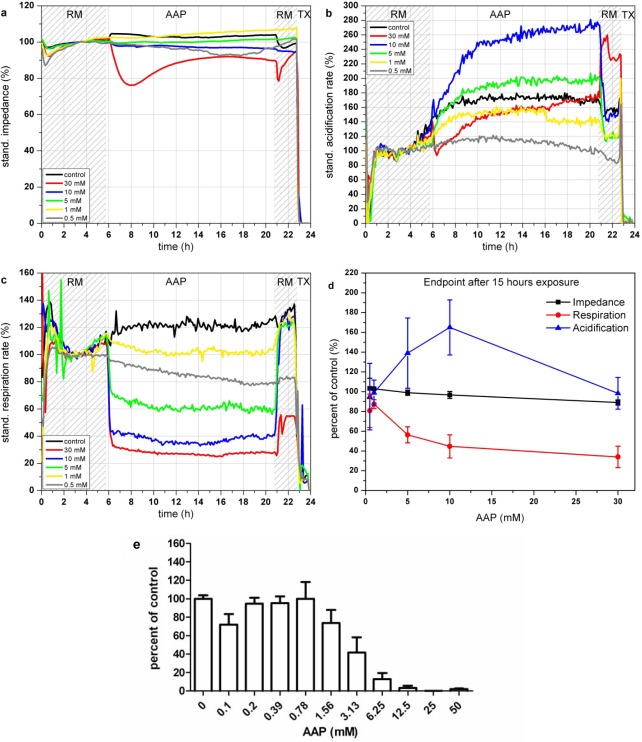
Time course of the impedance (**a**), acidification (**b**) and respiration (**c**) of L6 rat skeletal muscle cells during 24 h incubation with Acetaminophen in the Bionas 2500 Analyzing System. Vehicle control (black), 4.530 mg/L (30 mM, red), 1.510 mg/L (10 mM, blue), 755 mg/L (5 mM, green), 151 mg/L (1 mM, yellow) and 75.5 mg/L (0.5 mM, grey). Phases with running medium (RM) represent culture medium treatment without added substances. (**d**) Percent of control of impedance, respiration and acidification endpoint values measured after 15 h of AAP exposure (n = 3). (**e**) Results of BrdU assay of L6 cells incubated with AAP for 24 h.

AAP has an immediate effect on cellular respiration (see Figure 6(c)). Within 1 h after addition, AAP leads to a decrease of the respiration rate down to 30% for a concentration of 30 mM. Concentrations lower than 10 mM AAP show a fast recovery of the respiration (within 1 h) after 15 h of incubation. In contrast to the respiration, which shows a decrease for all AAP concentrations, the acidification shows a more complex signal pattern (see Figure 6(b)). Incubation with AAP concentrations lower than 5 mM results in a slight signal decrease compared to the control measurement. Higher concentrations (5–10 mM) activate the cell metabolism and lead to a strong increase of the acidification rates. The reduced capabilities of cells treated with 5–10 mM AAP to generate ATP via oxygen demanding oxidative phosphorylation, forces the cell to produce energy via increased glycolysis [46]. On the other hand, elevated levels of AAP (30 mM) lead also to a reduction of acidification. Excessive AAP toxification harms the cells permanently and therefore reduces all types of physiological activity. The impedance signals are constant over the whole tested concentration range (see Figure 6(a)). Concentrations of 30 mM lead to a decrease of 10–20% which is in good correlation with the reduced signals of the physiological parameters. Figure 6(d), showing the endpoint values of the changes relative to the control after 15 h of incubation with the various AAP concentrations, illustrates the opposing behavior of respiration and acidification at concentrations up to 10 mM.

The results of the BrdU proliferation assay are in accordance to the results obtained with the respiration parameter (see Figure 6(e)). In both cases, cytotoxic effects can be observed at concentrations of 1.0 mM AAP.

#### 3.2.4. Nicotine

Nicotine (3-substituted pyridine, C_10_H_14_N_2_) is an alkaloid present in several plants, especially in tobacco. Nicotine is a strong neurotoxin which was widely used as an insecticide in the past, and while nicotine itself is now forbidden, nicotine analogues are still heavily used. During tobacco processing and manufacturing tobacco products, nicotine which is a water soluble compound is transferred to the aqueous solution of the waste water [63]. Also tobacco dust with high content of nicotine can accumulate and nicotine can be extracted to ground waters [64]. In addition, the production of nicotine-based pharmaceuticals is another source of this substance that can arrive in municipal wastewater systems [65,66]. Nicotine plays an essential role in the development of lung cancer, as the incidence of cancer may be related to oxidative damage of DNA [67]. Furthermore, nicotine is believed to increase the generation of ROS which produces a condition of oxidative stress which is also able to result in the disruption of biological membranes and the oxidation of lipids and proteins. Exposure to nicotine leads to decreased activities of protective enzymes such as catalase and superoxide dismutase, causing lipid peroxidation of membrane lipids which are vital for the maintenance and integrity of cell function [68]. Interference with cellular protein synthesis and metabolism as well as reduced transmembrane potential has been observed [69,70].

**Figure 7.**
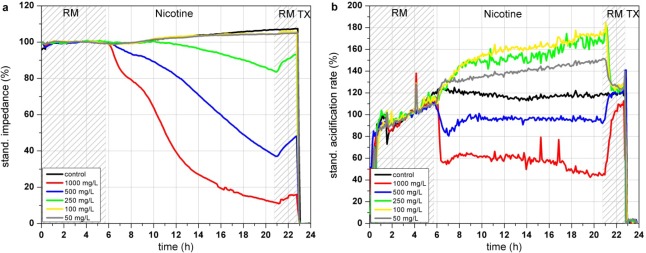

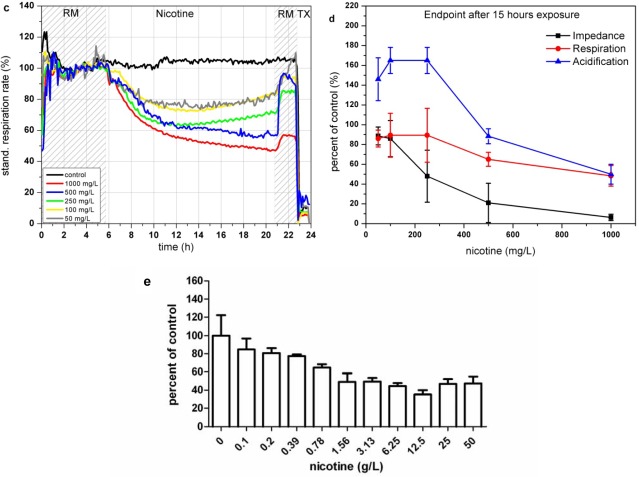
Time course of the impedance (**a**), acidification (**b**) and respiration (**c**) of L6 rat skeletal muscle cells during 24 h incubation with nicotine in the Bionas 2500 Analyzing System. Vehicle control (black), 1,000 mg/L (red), 500 mg/L (blue), 250 mg/L (green), 100 mg/L (yellow). Phases with running medium (RM) represent culture medium treatment without added substances. (**d**) Percent of control of impedance, respiration and acidification endpoint values measured after 15 h of nicotine exposure (n = 3). (**e**) Results of BrdU assay of L6 cells incubated with nicotine for 24 h.

In the Bionas analyzing system, nicotine shows a dose dependent decrease of impedance at high concentrations (>250 mg/L; see Figure 7(a)). This morphological disturbance is only slightly recovered after 3 h of incubation in clean running medium incubation. According to the literature, breakdown of the membrane integrity due to peroxidized lipid components in the cell membrane might be an explanation for the impedance changes. Concentrations up to 250 mg/L lead to a strong increase of cellular acidification while this trend is inverted at higher concentrations of 500 and 1,000 mg/L, resulting in a decreased metabolism (see Figure 7(b)). In contrast, the results of the respiration measurements are all oriented into the same direction (see Figure 7(c)). The endpoint values of the changes relative to the control after 15 h of incubation show that high concentrations of nicotine (500 and 1,000 mg/L) decrease the respiration down to 50–70% (see Figure 7(d)). One can assume that the generation of ROS causes damage in the respiration system of the cells. The BrdU assay results show a constant decrease of the proliferative activity at nicotine concentration range from ∼100 mg/L up to 12.5 g/L (see Figure 7(e)). The respiration rates obtained with the Bionas system and to a minor amount the impedance values are in accordance with the BrdU results as they show as well a continuously decrease at nicotine concentrations from 100 mg/L to 1,000 mg/L.

#### 3.2.5. Real Waste Water Sample

After conducting experiments with test substances to figure out the response behavior of L6 rat myoblasts, the cells were chosen to perform a close to application experiment. For this purpose L6 cells were incubated with a sample of industrial waste water (kindly provided by the Bavarian Environment Agency). The sample has been tested with standard wet chemistry tests (pH, conductivity, chloride, and sulfate) and biological toxicity tests (algae-, luminescent bacteria-, fish egg- and genotoxicity-assay, data not shown).

**Figure 8.**
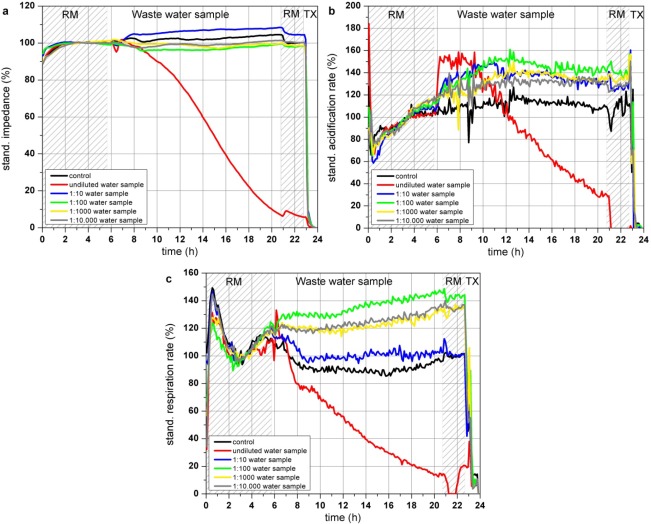
Time course of the impedance (**a**), acidification (**b**) and respiration (**c**) of L6 rat skeletal muscle cells during 24 h incubation with a real waste water sample in the Bionas 2500 Analyzing System. Vehicle control (black), undiluted (red), 1:10 (blue), 1:100 (green), 1:1,000 (yellow) and 1:100,000 (grey). Phases with running medium (RM) represent culture medium treatment without added substances.

Two hours after application of the undiluted water sample (Figure 8(a)), the impedance of confluent grown L6 cells started to decrease constantly whereas the dilution of the water sample between 1:100–1:10,000 only slightly effect the impedance of the cells. Right after introduction of the cells to the undiluted water sample the acidification rate increased with a subsequent decrease after 4 h, resulting in an acidification rate below 30% at the end of the experiment. Acidification rates of the cells incubated with dilutions of the water sample between 1:10–1:10,000 also showed an increase up to 140% but in these dilution ranges, the acidification rates stayed at that level (Figure 8(b)). Pollutants of this particular waste water sample might lead to an activated metabolism when they are supplied in a diluted form. Respiration of the cells was also reduced by the undiluted water sample starting rapidly after sample application. 14 h after the addition of the waste water sample the resulting respiration rate was lower than 20% of the start value, whereas the 1:10- and 1:100-dilution of the water sample showed similar effects as the acidification rates, as they are increased compared to the control measurement (Figure 8(c)).

This close to application experiment showed that cells exposed to an undiluted industrial waste water sample showed decreasing impedance and respiration rates. The acidification rate of the cells treated with the undiluted water sample increased at the first 4 h possibly to compensate a loss of energy of the almost immediately reduced cellular respiration. As the cells are further harmed by the water contaminations, the maximum of the metabolic activity is achieved and the metabolism totally collapsed after 4 h of sample incubation. Interestingly contaminant concentrations of the dilutions between 1:10 and 1:10,000 increased the metabolism and did not harm the cells like the undiluted water sample, as the acidification rates are raised up to approximately 130–140%. A strict dose response effect was not observed. One reason might be that the pollutants within the waste water sample might have opposite effects (activation and inhibition) on the cells, which can extinguish the observable signals of this sensor system as the signals show the sum of various pathways. At low initial pollutant concentrations or dilutions of high pollutant concentrations, the cells are able to compensate adverse effect on the cells by activating the metabolism and the respiration to gain sufficient energy. According to the standard biological and chemical characterization of the water, performed by the Bavarian Environment Agency, the sample was classified as moderate toxic and genotoxic. A toxic effect was clearly observed for the undiluted probe as all three cellular parameters are constantly decreased. Changes of the cell parameters are not a result of osmotic shock caused by pure water as the waste water probe was used only as a basis for a standard nutrition medium having the same osmolarity and nutrient composition like commercially available medium. It is likely that not only one but a number of different substances influence the cells at different cellular pathways and that depending on the substance the respiration or the impedance show more sensitive responses than the acidification.

## 4. Conclusions

The multiparametric readout of physiological and morphological cell parameters obtained with the Bionas SC 1000 Metabolic Chip is suitable for the monitoring of unknown toxic substances in water. In contrast to standard measurements performed with the Bionas System, this study demonstrated for the first time the use of a waste water sample as the basic liquid for the generation of cell culture medium. The main advantage of this application type is the possibility of the real-time monitoring of the aquatic environment by using the water as a cell culture medium basis. The method is operated in a label-free way without influencing the cells and is comparable or even more sensitive (e.g., Ni^2+^, Nicotine) with the results of a standard proliferation assay (BrdU). The system responds towards a wide range of potential contaminants, including heavy metals, neurotoxins and pharmaceuticals. The decision for an appropriate cell type is important as there are huge differences in the reaction patterns between the cells. As it is difficult to find one single cell line which is able to fulfill all criteria for the usage as a chemical sensor, an array of sensor chips, containing different cell types might be an appropriate solution. According to the instability of the control measurements, the gained results are just relative in reference to the control which has to be done parallel to every sample measurement. Therefore, changes of the signals can not be displayed as absolute values. A strict linearity in the dose-response behavior was not achieved. The main reason might be the fact, that there are numerous possibilities of eukaryotic cells to response towards a pollutant. The evaluated parameters are the sum of many intracellular pathways which can be activated or inhibited in multiple ways. Sometimes, the physiological parameters drift in opposite directions during the exposure towards low substance concentrations. The inhibition of one energy producing pathway seems to lead to an activation of other pathways in some cases to compensate the loss of energy. Detailed insight can only be obtained after further studies on the direct influence of the individual substance on the intracellular pathways at different concentrations. The use of three different sensor types at the same time on one sensor chip increases the chance that opposite effects (e.g., the presence of substances of stimulating and inhibiting effects in the water) are not canceled out for all three sensor signals. In addition, substances that only affect one of the three parameters can be still detected.

The purpose of the system is an overall indication of pollution and toxicity, rather than quantification of single substances. Further model substances and mixtures of theses substances have to be evaluated with different cells in ongoing experiments in order to improve the cell based sensor technology as a tool for environmental cytotoxicity sensors.

## Figures and Tables

**Table 1.** 33 hazardous and non-hazardous priority substances of the WFD [2].

Alachlor	Lead and its compounds	Cadmium and its compounds
Atrazine	Naphthalene	C_10–13_-chloroalkanes
Benzene	Nickel and its compounds	Endosulphan
Chlorfenvinphos	Octylphenols	Hexachlorobenzene
Chlorpyrifos	Pentachlorophenol	Hexachlorobutadiene
1,2-Dichloroethane	Simazine	Hexachlorocyclohexane
Dichloromethane	Trichlorobenzenes	Mercury and its compounds
Di(2-ethylhexyl)phthalate (DEHP)	Trichloromethane	Nonylphenols
Diuron	Trifluralin	Pentachlorobenzene
Fluoranthene	Anthracene	Polyaromatic hydrocarbons
Isoproturon	Pentabromodiphenylether	Tributyltin compounds

**Table 2.** Relative changes (in percent of normalized value after 3 h) of the cellular parameters of six evaluated cell lines after 18 h monitoring in the Bionas system.

**Cell type**	**Impedance**	**Acidification**	**Respiration**
HT-29	90%	130%	270%
Canine Hepatocytes	100%	90%	120%
HepG2	100%	95%	145%
L6	100%	100%	100%
NHDF	90%	110%	135%
V79	105%	190%	160%

**Table 3.** Limit of detection (LOD) of the tested substances (Ni, Cu, AAP, nicotine) after the treatment of L6 cells for 15 h.

**Substance**	**LOD Imp.**	**LOD pH**	**LOD O_2_**	**BrdU**	**German drinking water ordinance [53]**
**Ni^2+^ [mg/L]**	25	25	50	78	0.02
**Cu^2+^ [mg/L]**	-	-	5	6.25	2
**AAP [mM]**	-	10	10	3.13	No maximum value
**Nicotine [mg/L]**	500	100	1,000	1560	No maximum value
